# Medication adherence and its clinical and psychosocial correlates in inpatients with bipolar disorder in Vietnam: a cross-sectional study

**DOI:** 10.3389/fpsyt.2026.1819739

**Published:** 2026-09-03

**Authors:** Nhu Minh Hang Tran, Trung Thuc Le, Quang Ngoc Linh Nguyen, Vu Quoc Huy Nguyen

**Affiliations:** 1 Hue University of Medicine and Pharmacy, Hue University, Hue, Vietnam; 2 Central Psychiatric Hospital, No 2, Dong Nai, Vietnam

**Keywords:** bipolar disorders, family support, inpatients, insight into illness, medication adherence, Vietnam

## Abstract

**Background and objectives:**

Medication adherence remains a major challenge in the long-term management of bipolar disorder. Both clinical and psychosocial factors may be associated with adherence behavior, yet data from low- and middle-income countries remain limited. This study aimed to examine medication adherence and its clinical and psychosocial correlates among inpatients with bipolar disorder in Vietnam.

**Methods:**

A cross-sectional study was conducted among 90 inpatients diagnosed with bipolar disorder according to ICD-10 criteria at a central psychiatric hospital in Vietnam. Medication adherence was defined as taking ≥80% of prescribed medication during the maintenance phase. Demographic, clinical, and psychosocial variables were assessed. Univariate and multivariable logistic regression analyses were performed to identify factors associated with adherence.

**Results:**

Overall, 23.3% of participants met the criteria for medication adherence. Variables significant at p < 0.05 in univariate analysis were entered into a multivariable logistic regression model. In the final model, older age of onset (adjusted OR = 0.789, 95% CI: 0.673–0.925, p = 0.003), good insight into illness (adjusted OR = 46.189, 95% CI: 5.364–397.755, p < 0.001), and positive family support (adjusted OR = 53.930, 95% CI: 4.757–611.385, p = 0.001) were independently associated with adherence; perceived stigma was not independently associated with adherence after adjustment (adjusted OR = 0.329, 95% CI: 0.066–1.634, p = 0.174).

**Conclusion:**

Medication adherence was low in this inpatient sample. Psychosocial factors, particularly illness insight and family support, were more strongly associated with adherence than clinical characteristics. These findings highlight the importance of integrating psychosocial interventions into bipolar disorder management strategies in Vietnam.

## 1 Introduction

Bipolar disorder is a chronic and recurrent mood disorder characterized by episodes of mania, hypomania, and depression, with periods of full or partial remission between episodes (1). Although patients may appear clinically stable for intervals of time, the disorder carries a high lifetime relapse rate and requires long-term maintenance treatment to prevent recurrence and functional impairment (1, 2).

The episodic nature of bipolar disorder introduces a unique challenge: during symptom-free phases, many patients believe they no longer need medication. This tendency is exacerbated when insight into the illness is limited, leading to premature discontinuation of treatment. As a result, non-adherence remains one of the most significant barriers to effective long-term management, contributing to increased hospitalization, rate of relapse, suicide risk, treatment cost and to decrease quality of life (3). Many previous studies showed that poor medication adherence was high among patients with bipolar disorder (4–6).

While pharmacological factors such as side effects and treatment complexity have been widely discussed (7, 8), growing evidence indicates that adherence is strongly influenced by psychosocial dimensions. Both internalized and perceived public stigma can discourage treatment continuity (9). Family involvement plays an important role, particularly in collectivist societies, where relatives often supervise medication and provide emotional support (4, 7, 10). In parallel, clear communication and guidance from clinicians may help patients better understand the nature of bipolar disorder and the need for sustained treatment, especially during periods of mood stability (4, 11). Despite these insights, study on adherence in bipolar disorder remains limited in Vietnam. Cultural norms, family structures, and health communication practices may shape adherence patterns in ways distinct from Western contexts (4, 7). This study aimed to assess the prevalence of medication adherence and to identify its clinical and psychosocial correlates among inpatients with bipolar disorder in Vietnam.

## 2 Materials and methods

### 2.1 Study design and setting

This cross-sectional study was conducted at a Central Psychiatric Hospital, Vietnam, between April 2023 and July 2024. The study aimed to examine medication adherence and its clinical and psychosocial correlates among inpatients diagnosed with bipolar disorder.

### 2.2 Participants

All inpatients diagnosed with bipolar affective disorder according to the International Classification of Diseases, 10th Revision (ICD-10; code F31) were screened for eligibility.

#### 2.2.1 Inclusion criteria

Participants were eligible if they:

- Were aged 18 years or older;
- Had a confirmed ICD-10 diagnosis of bipolar affective disorder (F31);
- Agreed to participate and provided written informed consent.

#### 2.2.2 Exclusion criteria

Participants were excluded if they:

- Were pregnant or breastfeeding;
- Had severe physical illnesses that could interfere with assessment;
- Had mood disorders secondary to medical conditions or substance use.

A consecutive sampling strategy was applied: all inpatients admitted to the study site during the 15-month recruitment period (April 2023–July 2024) who met the eligibility criteria were invited to participate. Given the absence of prior local data on the prevalence of adherence and its correlates in this population, an a previous sample size calculation was not performed; the final sample of 90 represents all eligible admissions recruited during the study period.

Inpatients were selected because hospitalization provided a feasible setting for conducting structured face-to-face interviews with both patients and accompanying family members within a defined timeframe, and because medical records were readily accessible to verify clinical history and relapse episodes. We acknowledge that this restricts generalizability to outpatient populations, as noted in the Limitations.

Structured interviews were typically conducted approximately two weeks after admission, once patients had received initial treatment and acute symptoms (e.g., pronounced agitation, prominent psychotic features) had generally improved sufficiently to permit reliable communication. No formalized standardized readiness or capacity criterion was pre-specified to determine interview eligibility beyond the general exclusion criteria listed above; this is acknowledged as a limitation.

### 2.3 Measures

#### 2.3.1 Demographic, clinical, and psychosocial characteristics

Data were collected using structured interviews (both patients and close family member/caregivers) and medical record review.

Demographic variables included age, sex, educational level, and employment status.

Clinical variables included age at onset, duration of illness, number of relapse episodes, reported medication side effects and number of medications prescribed.

These psychosocial constructs were assessed using single-item structured questions developed through consensus among the study investigators and reviewed for face validity, given the absence of validated Vietnamese-language multi-item instruments for these constructs in the inpatient psychiatric setting at the time of data collection.

Before formal data collection, these questions were pilot-tested with 10 inpatients who were not part of the final study sample, to assess clarity and comprehensibility; wording was refined based on this pilot feedback before finalizing the interview protocol.

Psychosocial and treatment-related variables

- llness insight: Assessed via a structured single-item question regarding acknowledgment of having a mental disorder requiring treatment. Dichotomized as Yes/No.
- Medication beliefs: Assessed through structured questions regarding perceived necessity and perceived harm of medication. Categorized as positive versus negative beliefs.
- Family support: Defined as perceived emotional or practical support from family members in relation to treatment (e.g., reminders, accompaniment, encouragement). Dichotomized as Yes/No.
- Perceived stigma: Assessed via a structured single-item question asking whether the participant experienced teasing, judgment, discrimination, or unfair treatment due to mental illness during the past 12 months. The same question was also asked to a close family member to reduce potential misclassification. In cases of discrepancy, responses were adjudicated based on clinical judgment. Dichotomized as Yes/No.
- Health care guidance: Assessed based on participants’ perception of whether treatment instructions were sufficient and clear. Categorized as sufficient versus insufficient guidance.

#### 2.3.2 Medication adherence

Medication adherence was defined according to the American Medical Association criteria as taking ≥80% of prescribed medication during the maintenance phase (12) (≥3 months for manic episodes and ≥6 months for depressive episodes). The variable was coded as:

0 = Non-adherent
1 = Adherent

### 2.4 Statistical analysis

All analyses were performed using SPSS software (version 25.0).

#### 2.4.1 Descriptive analysis

Continuous variables were assessed for normality using the Shapiro–Wilk test and visual inspection of histograms and Q–Q plots.

- Normally distributed variables are presented as mean ± standard deviation (SD).
- Non-normally distributed variables are presented as median and interquartile range (IQR).
- Categorical variables are presented as frequencies and percentages.

#### 2.4.2 Univariate analysis

Univariate logistic regression analyses were conducted to examine associations between each independent variable and medication adherence.

Both continuous and categorical predictors were entered individually into logistic models. Odds ratios (ORs) and 95% confidence intervals (CIs) were reported.

#### 2.4.3 Multivariable logistic regression

Variables with p < 0.05 were entered into a multivariable logistic regression model using the enter method to identify independent correlates of medication adherence.

Adjusted odds ratios (aORs) with 95% confidence intervals were reported.

Model fit was evaluated using:

- Hosmer–Lemeshow goodness-of-fit test
- Nagelkerke’s R² to assess explanatory power

Multicollinearity was assessed using variance inflation factors (VIF), with VIF < 5 considered acceptable.

All tests were two-tailed, and p < 0.05 was considered statistically significant.

### 2.5 Ethics approval and consent to participate

This study was approved by the Biomedical Ethics Committee of Hue University of Medicine and Pharmacy (Approval No. H2023/399; issued April 20, 2023).

All participants provided written informed consent prior to enrollment. Participation was voluntary, and patients and/or their relatives were fully informed about the study objectives and procedures.

The study was conducted in accordance with the ethical principles outlined in the Declaration of Helsinki.

## 3 Results

### 3.1 Sample and demographic characteristics

A total of 90 inpatients diagnosed with bipolar disorder according ICD 10 criteria were included in the study. The gender distribution was relatively balanced, with 53.3% male and 46.7% female participants. The median age was 36 years (IQR: 29.75–44.25), indicating that the sample largely consisted of individuals in early to mid-adulthood (**Table 1**).

**Table 1:** Demographics in participants.

Characteristics	N=90
Gender (n,%)	
Female	42 (46.7)
Male	48 (53.3)
Age (years) (Median, IQR)	36 (29.75 – 44.25)
Level of education (n,%)	
Secondary or less	30 (33.3)
High school	40 (44.4)
University/college	20 (22.2)
Employment status (n,%)	
No	35 (38.9)
Stable/Unstable	55 (61.1)

Regarding educational level, nearly half of the participants had completed high school (44.4%), while one-third had secondary education or lower (33.3%). Only 22.2% had attained university or college level (**Table 1**).

In terms of employment status, 61.1% of participants reported having a stable or unstable job, whereas 38.9% were unemployed (**Table 1**).

### 3.2 Clinical and psychological characteristics

The mean age of illness onset was 29.06 ± 6.45 years. Participants had experienced a median of 6 relapse episodes (IQR: 5–8), and the median duration of illness was 6.0 years (IQR: 4.00–10.25) (**Table 2**).

**Table 2:** Clinical and psychological characteristics among participants.

Characteristics	N = 90
Age of onset (Mean ± SD)	29.06 ± 6,45
Number of relapses (Median, IQR)	6 (5 – 8)
Duration of disorders (years) (Median, IQR)	6.00 (4.00 – 10.25)
Number of medication prescribed (n,%)	
< 3	28 (31.1)
≥ 3	62 (68.9)
Having side effects of medication (n,%)	
Yes	65 (72.2)
No	25 (27.8)
Insight about disorder (n,%)	
Yes, I have disorder and need being treated	37 (41.1)
No, I have no disorder	53 (58.9)
Medication beliefs	
Negative	67 (74.4)
Positive	23 (25.6)
Family support	
Yes	50 (55.6)
No	40 (44.4)
Health care guidance	
Sufficient and careful	43 (47.8)
Insufficient/no guidance	47 (52.2)
Perceived stigma	
Yes	67 (74.4)
No	23 (25.6)

Polypharmacy was common, with 68.9% of participants receiving three or more medications. A high proportion (72.2%) reported experiencing medication side effects (**Table 2**).

Insight into illness was limited, as only 41.1% acknowledged having a mental disorder requiring treatment, while 58.9% denied having any disorder. Negative medication beliefs were prevalent (74.4%), and only 25.6% reported positive beliefs toward pharmacotherapy (**Table 2**).

More than half of the participants reported receiving family support (55.6%), while 44.4% did not. Health care guidance was reported as sufficient and careful by 47.8% of participants, whereas 52.2% reported insufficient or no guidance. Perceived stigma was prevalent, affecting 74.4% of the sample.

### 3.3 Medication adherence in participants

Among the 90 participants, 21 patients (23.3%) met the criteria for medication adherence, whereas 69 patients (76.7%) were classified as non-adherent (**Table 3**).

**Table 3:** Prevalence of medication adherence among patients with bipolar affective disorder.

Characteristics	n,%
Adherence	21 (23.3)
Non - adherence	69 (76.7)

### 3.4 Factors associated with medication adherence in participants

In univariate logistic regression analysis, age of onset was significantly associated with medication adherence (OR = 0.913, 95% CI: 0.838–0.994, p = 0.036). Perceived stigma was also significantly associated with adherence (OR = 0.339, 95% CI: 0.119–0.965, p = 0.043) (**Table 4**).

**Table 4:** Factors associated with medication adherence among participants (univariate logistic regression analysis).

Characteristics	Exp (B)	95% CI	P – value
Gender	1.348	0.507 – 0.3589	0.55
Age	0.955	0.906 – 1.006	0.086
Level of education	1.645	0.839 – 3.227	0.147
Employment status	0.552	0.191-1.593	0.272
Age of onset	0.913	0.838 – 0.994	0.036
Number of relapses	0.939	0.802 – 1.098	0.428
Duration of disorders	0.960	0.880-1.048	0.361
Number of medication Prescribed	0.441	0.133-1.460	0.180
Having side effects of medication	1.417	0.494– 4.066	0.517
Good Insight about disorder	5.347	1.824 - 15.625	0.002
Medication beliefs	0.817	0.274 – 2.440	0.718
Positive Family support	26.000	3.301 – 204.812	0.002
Health care guidance	0.363	0.130-1.011	0.052
Perceived stigma	0.339	0.119-0.965	0.043

Results in **Table 4** showed that insight into illness was a factor associated with adherence (OR = 5.347, 95% CI: 1.824–15.625, p = 0.002). In addition, family support demonstrated a positive association with medication adherence (OR = 26.000, 95% CI: 3.301–204.812, p = 0.002).

Health care guidance showed a borderline association with adherence (OR = 0.363, 95% CI: 0.130–1.011, p = 0.052). (**Table 4**).

Other demographic and clinical variables, including gender, education level, employment status, number of relapses, duration of disorder, number of prescribed medications, side effects, and medication beliefs, were not significantly associated with adherence (all p > 0.05) (**Table 4**).

### 3.5 Multivariable logistic regression analysis

After adjusting for potential confounders, age of onset, insight into illness and family support remained independently associated with medication adherence (**Table 5**).

**Table 5:** Multivariable logistic regression analysis.

Characteristics	aOR	95% CI	P - value
Age of onset	0.789	0.673-0.925	0.003
Good Insight about disorder (1)	46.189	5.364 – 397.755	<0.001
Positive Family support (1)	53.930	4.757 – 611.385	0.001
Perceived stigma (1)	0.329	0.066 – 1.634	0.174

Participants with good illness insight were significantly more likely to adhere to treatment compared to those without insight (aOR = 46.189, 95% CI: 5.364–397.755, p < 0.001). Similarly, positive family support was independently associated with higher adherence (aOR = 53.930, 95% CI: 4.757–611.385, p = 0.001), The older the age of onset, the poorer the likelihood of adherence to treatment (aOR=0.789, 95% CI:0.673-0.925). (**Table 5**) perceived stigma, was not statistically significant in the adjusted model (**Table 5**).

The wide confidence intervals observed for insight and family support indicate limited precision of effect size estimates (**Table 5**).

## 4 Discussion

### 4.1 Prevalence of medication adherence in the sample

In this cross-sectional study of inpatients with bipolar disorder in Vietnam, medication adherence was low, with only 23.3% of participants meeting adherence criteria. The low adherence rate observed in our study is consistent with previous findings from both Vietnam and other countries, although methodological differences should be considered.

In Vietnam, Lê Thị Thanh Huyền reported 36.4% poor adherence, 19.8% moderate adherence, and 43.8% good adherence (4). Notably, a high proportion of patients reported forgetting medication within the previous two weeks or experiencing difficulty remembering all prescribed medications.

International studies have reported similar patterns. Belete et al. found that 68.8% of patients demonstrated low to moderate adherence, with only 31.2% categorized as having good adherence (5). Okasha reported that 58% of patients had poor adherence when assessed using MARS (13). In India, Selvakumar et al. observed that 60.6% of patients were classified as having low adherence (14). Across these diverse settings and assessment tools, optimal adherence rarely exceeds 50%.

Scott et al. assessed adherence using the Tablet Routines Questionnaire and plasma drug levels (6). Approximately one-third of patients reported partial adherence, and subtherapeutic plasma levels were common. Importantly, partially adherent patients with low plasma levels had significantly higher psychiatric hospitalization rates over 18 months compared to fully adherent patients. These findings highlight the clinical relevance of adherence assessment and its impact on long-term outcomes.

### 4.2 Demographic characteristics and medication adherence

In our study, medication adherence was not significantly associated with age, sex, employment status, or educational level. These findings suggest that sociodemographic characteristics may not be primary determinants of adherence behavior in this inpatient population. Similar results have been reported in previous research. A review by Jawad et al. examining medication adherence in bipolar disorder in the United States concluded that demographic and socioeconomic factors were not major determinants of adherence (3). Sajatovic et al. (2011) found no significant gender differences in medication adherence among patients with bipolar disorder (15). More recently, Sajatovic and colleagues (2023), using a definition of non-adherence comparable to ours (≥20% missed medication), also reported that age and other demographic attributes were not significantly associated with adherence among adolescents and young adults with bipolar disorder (16).

However, findings across studies remain inconsistent. Okasha et al. (2020), in a one-year longitudinal study in Egypt, reported higher adherence among older, married, female, and more highly educated patients (13). In contrast, Bauer et al. (2019) observed that female sex was associated with non-adherence (17). Selvakumar et al. (2018) found that employment was associated with better adherence (OR = 2.78, 95% CI: 1.02–7.59) (14). These discrepancies may reflect differences in study design, sample size, cultural context, and methods used to assess adherence.

As noted by Chakrabarti et al. (2016), early research on non-adherence in bipolar disorder primarily focused on demographic and clinical factors. However, inconsistent findings and the complex, multidimensional nature of adherence behavior suggest that demographic characteristics alone are insufficient to explain or predict medication non-adherence (18).

Our findings support the view that adherence in bipolar disorder is unlikely to be determined by static sociodemographic variables alone and may instead be more strongly associated with psychological and relational factors.

### 4.3 Clinical characteristics and medication adherence

In the present study, most clinical variables, including duration of illness, number of relapse episodes, number of prescribed medications, and reported side effects were not independently associated with medication adherence after multivariable adjustment (**Tables 4**, **5**). These findings suggest that illness-related characteristics alone may not adequately explain adherence behavior in hospitalized patients with bipolar disorder. Age of onset was an exception, in our final multivariable model, older age of onset was independently associated with poorer adherence (adjusted OR = 0.789, 95% CI: 0.673–0.925, p = 0.003).

Previous studies examining clinical predictors of adherence in patients with bipolar disorder have reported inconsistent results. Bauer et al. (2019) found no significant association between medication adherence and age of onset, medication type, or number of prescribed agents (17). However, other studies have suggested that treatment complexity may play a role. Jawad et al., Fung et al., and Lê Thị Thanh Huyền reported that polypharmacy and complex medication regimens were associated with poorer adherence (3, 4, 19). In particular, Fung et al. (2019) demonstrated that polytherapy was significantly associated with non-adherence (OR = 2.51, 95% CI: 1.81–3.50) (19). Similarly, Lê Thị Thanh Huyền found that a notable proportion of patients discontinued medication due to regimen complexity and difficulty remembering multiple prescriptions (4).

Hospitalization history has also been examined as a clinical correlate. Corréard et al. observed that fewer prior hospitalizations were associated with higher adherence in both younger and older patients (20). Consistently, Lintunen et al. (2023) reported increased risk of non-adherence among patients with more frequent hospitalizations and shorter illness duration (21). These findings suggest that illness course characteristics may influence adherence, although results are not consistent across settings.

Medication side effects have been frequently cited as barriers to adherence. Jawad et al. identified adverse effects as an important factor associated with non-adherence (3). Corréard et al. similarly reported that greater side-effect burden predicted lower adherence across age groups (20). A review by Mago et al. further emphasized that adverse effects are among the most commonly reported reasons for discontinuation of mood stabilizers in bipolar disorder (22).

The literature suggests that clinical determinants of adherence such as regimen complexity, hospitalization history, and side-effect burden may contribute to medication-taking behavior, although findings remain inconsistent. This variability likely reflects differences in study design, patient populations, healthcare systems, and methods used to define and measure adherence.

### 4.4 Psychological factors associated with medication adherence

Illness insight showed the largest point estimate among the independent correlate of medication adherence in our study (**Table 5**) although the wide confidence interval warrants cautious interpretation. Beyond simple acknowledgment of diagnosis, insight reflects a broader cognitive and emotional appraisal of illness, including perceived severity, perceived need for treatment, beliefs about personal control, and expectations regarding relapse.

A growing body of literature supports the central role of illness awareness in treatment adherence among individuals with bipolar disorder and related psychiatric conditions. Adams et al. demonstrated that highly adherent and partially adherent patients differed significantly in their perceptions of illness severity, beliefs about self-control over the disorder, and concerns about rehospitalization (23). These findings suggest that adherence is closely tied to how patients cognitively represent their illness rather than merely to symptom burden.

Similarly, Jawad et al. identified poor illness understanding as one of the key factors associated with non-adherence in bipolar disorder (3). Alcala et al. further showed that perceived need for treatment during acute episodes, illness recognition, shared clinical decision-making, and attribution of symptoms to biological causes were important motivational drivers of pharmacological treatment engagement (24). These elements collectively highlight that adherence behavior is strongly influenced by patients’ explanatory models of illness.

Qualitative research reinforces this perspective. Clatworthy et al. reported that intentional non-adherence was often linked to doubts about the personal necessity of medication, rejection of diagnosis, beliefs that the disorder was not chronic, or perceptions that the condition was uncontrollable (25). Such findings suggest that non-adherence may reflect a coherent belief system rather than simple forgetfulness or negligence.

These studies indicated that insight in bipolar disorder was multidimensional and deeply embedded in patients’ beliefs about illness identity, chronicity, and controllability. In the Vietnamese cultural context, where mental illness may be interpreted through psychosocial or spiritual frameworks, limited biomedical attribution may further weaken perceived necessity for long-term pharmacotherapy. Therefore, the strong association observed in our study likely reflects not only symptom-related cognitive impairment but also culturally shaped illness representations. These findings underscore the importance of structured psychoeducation, culturally sensitive communication, and shared decision-making in enhancing adherence. Addressing illness perceptions may be as critical as optimizing pharmacological regimens in promoting sustained treatment engagement.

In univariate analysis, perceived stigma was associated with medication adherence; however, this association did not remain statistically significant after adjustment for other psychosocial factors. This finding suggests that while stigma may be related to adherence behavior, its effect may not be independent of other variables such as illness insight and family support.

Previous research has highlighted the potential role of stigma in shaping treatment engagement in severe mental disorders (18, 26, 27). Perceived or internalized stigma may reduce help-seeking behavior, weaken illness acceptance, and negatively influence attitudes toward long-term pharmacotherapy. Concerns about social judgment, labeling, and discrimination can discourage patients from openly acknowledging their condition or consistently adhering to medication.

However, the relationship between stigma and adherence appears to be complex. It is possible that stigma operates indirectly associated with illness insight or perceived need for treatment. Patients who internalize stigmatizing beliefs may be more likely to deny illness or minimize its severity, thereby reducing motivation for sustained pharmacological treatment. When these cognitive and relational variables are accounted for in multivariable models, the independent effect of stigma may diminish.

In the Vietnamese cultural context, where mental illness can carry moral and familial implications, perceived stigma may still represent an important social stressor. Nevertheless, our findings suggest that stigma alone may not independently determine medication adherence once psychological and family-related factors are considered.

In univariate analysis, healthcare guidance showed a marginal association with medication adherence; however, this association did not remain statistically significant in the multivariable model. This suggests that while the adequacy and clarity of professional guidance may be associated with adherence behavior, though this association may not be independent of other psychological and relational determinants.

The role of healthcare professionals in promoting adherence has been widely emphasized. Jawad et al. identified poor therapeutic alliance as an important factor associated with non-adherence and recommended that clinicians routinely inquire about missed medication in a non-judgmental manner (3). Interventions such as shared decision-making, targeted psychoeducation, and practical reminder strategies have been suggested as feasible approaches to improve adherence.

Interventional studies further support this perspective. Lama et al. reported significant improvements in adherence following structured psychiatric nursing interventions (11). Motivational interviewing has also shown promising results. McKenzie et al. (2015) demonstrated that a brief motivational interviewing intervention significantly improved medication adherence, self-efficacy, and motivation for change among outpatients with bipolar disorder (28). Similar findings were reported by Pakpour et al. (2017) (29). These findings suggest that adherence is modifiable and may respond to structured communication-based interventions.

While healthcare guidance was not an independent predictor in our multivariable model, evidence from interventional studies indicates that strengthening therapeutic alliance and patient-centered communication may play a crucial role in improving adherence outcomes.

Regarding family support, our results showed that family support was independently associated with medication adherence in our multivariable analysis, highlighting the importance of relational factors in the management of bipolar disorder. Patients who reported supportive family involvement were significantly more likely to adhere to prescribed treatment compared with those who did not report such support.

The role of family involvement in adherence has been documented in both Vietnamese and international research. Lê Thị Thanh Huyền reported that a proportion of patients discontinued medication due to lack of family support, suggesting that the absence of active engagement from family members may directly contribute to treatment interruption (4). Similarly, Chang et al. demonstrated that higher levels of perceived social support and stronger beliefs that health outcomes are influenced by significant others such as family members or clinicians were associated with more positive attitudes toward medication (30). A synthesis by Prajapati et al. further identified social influence, including family support, as a key determinant of adherence in bipolar disorder (10). Importantly, supportive behaviors, such as encouraging medication use and monitoring symptoms facilitated adherence, whereas discouragement or ambivalence from family members functioned as barriers.

In the Vietnamese context, family involvement often extends beyond emotional encouragement to active behavioral regulation. Family members commonly accompany patients to follow-up appointments, supervise medication storage, provide daily reminders, and directly monitor medication intake (7). In some cases, relatives assume partial responsibility for treatment management, particularly when patients exhibit limited illness insight or fluctuating mood symptoms. Such practical involvement may reduce forgetfulness, enhance accountability, and reinforce perceived necessity of treatment. These culturally embedded caregiving practices may partly explain the strong association observed between family support and medication adherence in our sample. In collectivist societies, adherence may therefore reflect a shared family-driven process rather than solely an individual-level decision.

At the same time, the magnitude of association observed in our study should be interpreted with caution. The adjusted odds ratio for family support was accompanied by a wide 95% confidence interval, indicating limited statistical precision. This imprecision is likely related to sample size and the distribution of adherence outcomes. Therefore, while the direction of association appears consistent with previous literature and theoretically plausible, the exact strength of the effect cannot be estimated with high certainty.

Moreover, family support was assessed using a single self-reported dichotomous measure, which does not capture the multidimensional nature of family functioning. Support may vary in intensity, consistency, and quality, and family dynamics can also include controlling or conflictual elements that were not evaluated in this study.

Given the exploratory nature of this study and the imprecision of the effect estimates, these findings should be regarded as hypothesis-generating rather than confirmatory, and require replication in larger, adequately powered samples.

## 5 Strengths and limitations

This study has several strengths. First, it examined both clinical and psychosocial correlates of medication adherence within the same analytic framework, allowing for a comprehensive evaluation of potential determinants. By retaining key clinical variables as continuous measures rather than arbitrarily categorizing them, the analysis preserved statistical information and reduced misclassification bias. Second, the inclusion of culturally relevant psychosocial constructs such as family support and perceived stigma provides context-specific insights into adherence behavior in Vietnam. Third, the use of multivariable logistic regression enabled identification of independent correlates while accounting for potential confounding among psychological and relational variables.

Nevertheless, several limitations should be acknowledged. The cross-sectional design precludes causal inference, and the directionality of associations cannot be definitively established. The relatively modest sample size may have contributed to wide confidence intervals in the multivariable model, particularly for psychosocial predictors, thereby limiting the precision of effect estimates. In addition, several psychosocial variables were assessed using single-item, non-validated measures. Although operational definitions were clearly specified, these measures may not fully capture the multidimensional nature of constructs such as family support or perceived stigma. Because these single-item measures were not formally validated, reliability could not be quantified, and comparability with studies using standardized multidimensional instruments (e.g., validated insight or stigma scales) is limited. This may have introduced non-differential measurement error, potentially biasing associations toward or away from the null.

As no a previous sample size calculation was performed, the study may have been underpowered to produce stable and precise multivariable estimates, particularly for psychosocial variables with skewed distributions. Relatedly, the adjusted odds ratios for illness insight (46.189) and family support (53.930) were unusually large and accompanied by wide confidence intervals, consistent with sparse-data bias arising from small cell counts in the underlying cross-tabulations, a recognized limitation of maximum-likelihood logistic regression in modest samples. These estimates should therefore be interpreted as indicating a strong directional association rather than a precise quantitative effect size.

Retrospectively, a sample of 90 provides an estimate of adherence prevalence (23.3%) with a 95% confidence interval margin of error of approximately ±8.7 percentage points, which is acceptable for an exploratory study in a population with no previous local data. In response to reviewer feedback, we revised our variable selection strategy, only variables significant at p<0.05 in univariate analysis were entered into the multivariable model, reducing the model from 8 to 4 candidate predictors (age of onset, perceived stigma, family support, and insight into illness). Applying the events per variable (EPV) criterion to this revised model (21 adherent participants across 4 predictors, EPV ≈ 5.25) remains below the conventional threshold of EPV ≥ 10, but represents a substantial improvement over the original 8 variable model (EPV ≈ 2.6) and is consistent with commonly cited minimum thresholds (EPV ≥ 5) proposed in the methodological literature for exploratory models. The 95% confidence intervals for insight into illness and family support narrowed accordingly (from 7.54–1293.38 to 5.36–397.76, and from 5.53–988.79 to 4.76–611.39, respectively) but remain wide, which we attribute to the low number of adherent participants (n=21) relative to the strength of these associations rather than to model overfitting from an excessive number of predictors.

This study was conducted at a single psychiatric hospital among hospitalized patients only; findings may not be generalizable to outpatients or community dwelling individuals with bipolar disorder, who may differ in illness severity, psychosocial circumstances, and access to family or clinical support. Because only inpatients were enrolled, the sample likely over represents individuals with more severe or acute presentations who required hospitalization, who may differ systematically in adherence behavior, insight, and family involvement; this may have introduced selection bias that should be considered when interpreting the reported associations.

Future longitudinal studies using larger samples and validated multidimensional instruments are warranted to clarify causal pathways and refine intervention targets.

## 6 Conclusion

In this cross-sectional study of inpatients with bipolar disorder in Vietnam, medication adherence was low, with fewer than one-quarter of participants meeting predefined adherence criteria. While multiple demographic and clinical variables were examined, psychosocial factors, particularly illness insight and family support were independent correlates of adherence.

These findings suggest that medication-taking behavior in bipolar disorder may be more strongly associated with cognitive and relational processes than with demographic characteristics or illness-related indicators. In the Vietnamese cultural context, where family structures are highly interdependent, adherence should be understood not only as an individual behavior but also as a socially embedded process.

Although causality cannot be inferred, the results underscore the importance of integrating culturally sensitive psychoeducation, family-inclusive care, and patient-centered communication strategies into routine management of bipolar disorder. Future longitudinal research using larger samples and validated multidimensional measures is needed to clarify causal pathways and refine intervention approaches.

## Data Availability

The raw data supporting the conclusions of this article will be made available by the authors, without undue reservation.
